# Nursing students’ perspectives on clinical education

**Published:** 2015-01

**Authors:** MOHAMMAD REZA HEIDARI, REZA NOROUZADEH

**Affiliations:** Nursing and Midwifery School, Shahed University, Tehran, Iran

**Keywords:** Education, Nursing, Perception, Students

## Abstract

**Introduction: **The importance of optimal clinical nursing education in professional skills development is undeniable. In clinical education, nursing students are often faced with problems. Recognizing nursing students’ perception on clinical education is the first step to remove the barriers of this challenge.

**Methods **This descriptive cross-sectional study was conducted to determine the nursing students’ perspectives on clinical education. 150 nursing students were selected randomly from nursing and midwifery schools (Tehran). Data collection instrument was a researcher made questionnaire consisting of five domains: objective and curricula, instructor, feedback to student in clinical field, clinical environment, supervision and evaluation. Mean and standard deviation were calculated for each item, using SPSS, ver.14. Chi- square test was used to compare the nursing students’ perspectives on clinical education based on age, sex and the work experience. The significance level was considered 0.05.

**Results: **Mean age of the students was 21.58±26.97 students (66%) were male. 44 students (30.1%) had work experience (3.58±6.48 month). Male and female students had different perceptions in domains of clinical education (p<0.05). Nursing student had different perceptions as to objectives and curricula (p=0.039), how to deal with students in the clinical environment (p=0.032), supervision, and evaluation (p<0.001) with respect to their work experience duration. The most positive responses were in clinical instructor (81.5%) and the most negative ones were the clinical environment (33.66%), respectively.

**Conclusion: **Providing an optimal clinical environment and improving the supervision and evaluation of student practice should prioritized in schools of nursing and midwifery.

## Introduction

Clinical teaching in is an important part of nursing education (1). Understanding the factors that influence the quality and quantity of clinical education is helpful in solving the related problems (2). Nurses are repeatedly exposed to situations that may cause them to suffer and reduce their ability to serve the patients (3). Therefore, assessment of clinical skills by undergraduate nursing programmers is important to be considered (4). The importance of clinical nursing education is undeniable in personal, professional and clinical skills development (5). Through nursing education, nursing students will be able to obtain necessary knowledge and skills to assist public health (6). Success in this subject requires the adoption of effective approaches to familiarize the students with new knowledge and essential needs of the clients (7). The existing clinical education does not meet the clinical skills competence of the students. Because of the large gap between theoretical and clinical nursing education, recognition of clinical education problems is important (5, 8, 9).  Due to their immediate attendance in this process, the students are the best and most reliable sources for detection of these problems (10). Some studies have proposed the clinical education challenges from the students’ perception, such as uunavailability of qualified instructors, lack of sufficient facilities, lack of cooperation of the staff in clinical education, lack of procedures to be practiced by students, lack of clinical education tasks, mismatch between objectives and the content of clinical education curriculum, lack of acquisition of prerequisites before entering the clinical fields, lack of appropriate opportunities for learning, lack of motivation among students, knowledge deficit about the profession, implementation of learned objectives in clinical setting , students' lack of awareness about the objectives and evaluation methods (11-15). Given the central role of students in clinical education, understanding their attitude toward the implementation of this method of education is necessary. To obtain a reliable finding, this multicenter research was conducted with special focus on perceptions of clinical education among senior nursing students.

## Methods

This is a descriptive cross-sectional study aiming to determine the nursing students’ perspectives on clinical education (2012-2013). Stratified sampling was done on bachelor nursing students who entered the clinical environment. The samples were selected from each school (Tehran University of Medical Sciences, Islamic Azad University, Baqiyatallah University of Medical Sciences, Army University of Medical Sciences, Shahed University, Shahid Beheshti University of Medical Sciences). After a pilot study on 15 students, by taking the mean of 11.21, standard deviation of 1.72 and a maximum sampling error (d=0.02), 150 students were selected. Data collection instrument included a researcher made questionnaire consisting of 31 questions in Likert style ranking (yes, somewhat, and no) in five domains: objectives and curriculum, clinical instructor, how to deal with students in clinical settings, supervision and evaluation. Content and face validity was evaluated and confirmed by 10 nursing faculty academic members. Cronbach's alpha =93% was obtained for 20% of the samples. Between the two phases of questionnaire completion, the interclass correlation coefficient (ICC) was 0.556. All ethical issues including  obtaining the approval of research council of  Shahed University, introducing the researcher to the subjects, allowing voluntary participation, and informing the students about the research objectiveswere considered. Also, the participants were assured that all the information would be confidential. To identify clinical education status, the percentages of negative and positive percpectives were extracted. The mean and standard deviation of each domain of the questionnaire was calculated. Chi-square test was used to compare the clinical education for nursing students’ perspective according to their socio-demographic characteristics (age, gender, work experience in the clinical settings and the working time). Non-parametric tests (Mann-Whitney and Kruskal-Wallis) were used to compare the level of students' perspectives on clinical education in each of the domains. The statistical analysis was performed through SPSS 14 ( SPSS Inc, Chicago ,IL ,USA). The significance level for analyses was set at α=5. 

## Results

The mean age of the students was 21.7±1.68 years (range: 19-28 years). 66% (n=97) of them were male. Among the students, 30.1% (n=44) had an experience of student work in hospital. The mean work time was 3.58 ± 0.48 months. In the domain of clinical educational objectives, the most positive scores (agreement) were related to "presenting job description to the student " and "providing clinical education objectives on the first day of education" with 54% of responses for each item, and the lowest score (disagreement) were "Coordination between educational objectives and expectations of staff from students " belonged to 33.6% of responses. The mean percentage of positive responses (agree view) was in the domain of educational objectives (44.24%) and for negative responses (opposing views) it was 22.41%. In domain of instructor, the most score (agree views) was "expectation of clinical instructor to students’ on time attendance at the field"(74.5%) and the lowest score (opposing views) belonged to "clinical instructor in dealing with the problem reduces the students’ stress "(22.1%). In domain of clinical instructor, 79.53% of responses were positive (agree views) and 18.5% negative (opposing views). In domain of interaction with students, the most score (agree views) was related to" strengthen the self-assurance of students by the instructor and staff" (46.6%) and the lowest score (opposing view) was given to "empowering the students to make decisions in patient care planning "(25.7%). In domain of interaction with students, 39.75% of the responses were positive (agree views) and 22.53% negative (opposing views). In domain of clinical environment, the most score (agree views) was related to "sufficient number of patients for learning" (45.9%) and the lowest one (opposing views view) belonged to "motivation for occupation through clinical environment" (72.2%). In domain of clinical environment, 35.3% of responses were positive (agree views) and 33.66% negative (opposing views). The most positive responses (agree views) was related to clinical instructor (81.5%) and the most negative ones (opposing views) to clinical environment (33.66%) (Table 1).

**Table1:** The students' perspective about clinical education domains

**Domains of clinical education**	Positive responses N (%) ****	Negative responses N (%) ****
Education objectives	114 (77.59%)	36 (22.41%)
Instructor	123 (81.50%)	27 (18.50%)
How to deal with students in clinical settings	117 (77.47%)	33 (22.53%)
Clinical environment	114 (66.34%)	36 (33.66%)
Evaluation and supervision	116 (76.85%)	34 (23.15%)

Male and female students had different perspectives in learning objectives (p=0.001), instructor (p=0.046), how to deal with students in clinical settings (p=0.005), clinical environment (p=0.001), evaluation and supervision (p=0.005). Also, male students were more positive than females in all domains of clinical education. The perspective of clinical education did not differ by age or work experience. But, nursing students had differ perspective on educational objectives, (p=0.039), monitoring/evaluation (p<0.001), and interacting with students (p=0.032) according to the duration of their work. The mean of supervision and evaluation decreased with increase in the work experience (p=0.002) (Table 2).

**Table2:** The students' perspective of clinical education domains by gender and work years

**Domains of clinical education**	**Gender**	**Mean rank**	***p***
Learning objectives	Male	79.75	0.001
	Female	56.17	
Instructor	Male	75.82	0.046
	Female	61.35	
How to deal with students in clinical settings	Male	79.86	0.005
	Female	59.15	
Clinical environment	Male	79.09	0.001
	Female	54.29	
Evaluation and supervision	Male	79.15	0.005
	Female	59.60	
Evaluation and supervision	<6 month	60.96	0.002
	6-12 month	43.78	
	>12 month	38.97	

## Discussion

Findings showed that most students believed that tasks and objectives are presented at the beginning of clinical education. However, there were low ratings to educational objectives and staff expectations of students; it means a challenge or an obstacle in these items. Accordingly, in Delaram’s study (2013), students have outlined discrepancy between the staff expectations and objectives of clinical education (15).

Similarly, in Khadivzadeh’s research (2004), most of the students considered the clinical education objectives as clear and consistent with the content of clinical education (16). In contrast to our findings, Taghinejad (2008), Zaighami (2004) and Ebrahami (2004) stated that nursing students have considered the lack of clear job description and inappropriateness clinical education with objectives as the major problems in clinical education (5, 11, 12). In the instructor domain, the most positive responses were related to instructor expectation of timely students’ attendance at clinical field and the most negative responses belonged to the ability to reduce the students’ stress in dealing with problems. Similarly, Delaram (2013) suggest students’ timely attendance as the strengths of clinical education from the students’ perspective (15).

In some studies, different opinions are presented about the clinical instructor characteristics. For example, in the study conducted by Ebrahimi (2004) the lack of support and insufficient justification for nursing profession were clinical education barriers related to the instructor (12). Gignac-Caille (2002) showed that the students' interpersonal relationships as the clinical capabilities can be effective in the performance of clinical instructors (17). The findings of this study showed that most of the students responded c appropriately with the instructor and were supported to increase the ability for dealing with stress in clinical practice. But, Tavakoli Ghochani (2009) in determining the characteristics of an effective clinical instructor revealed that the students had given lower scores to interpersonal interaction by the instructor (18).

Fakhr Movahedi’s in his study (2013) showed that nursing students scored the clinical instructors’ behaviors above average (1). In domain of “how to deal with students in the clinical environment”, most of the students responded positively to reinforcing self-assurance by the instructor and staff and the most negative responses were given to authority to make decisions to planning patient care. Similar to this finding, Delaram (2013) showed students are not authorized to make decisions in patient care at clinical education (15). Also, Mohammadi (2004) described the most important problems as the lack of cooperation and improper communication with students in clinical education (19).

Accordingly, “sufficient number of patients for learning” was the strength and “effectiveness of motivation for future jobs” was at a disadvantage from the nursing students’ perspective. In other studies, the lack of the necessary educational aids, great number of students and forcing students to perform the duties of the staff, the lack of adequate facilities, lack of possible implementation learned items in clinical environment were the most negative points from the nursing students’ perspective (16, 19, 20). Henderson (2006) investigated the students' perception of the psycho-social clinical learning environment and showed that the stable clinical environment can support further learning and impart more knowledge to students in clinical education (21).

Payne and Glaspie (2013) found that students' performance is not influenced by the perception of the clinical learning environment. In other words, a well-accepted clinical education environment may not have significant effect on the student outcomes (22). O'Mara (2013) studied the nursing students' experiences of challenges related to the clinical learning environment. Nursing students perceived these challenges in relation to clinical environment and learning experiences, so it has affected their learning opportunities and professional identity (23).

According to the findings, the least ratings were monitoring and evaluation (complete supervision on clinical education and giving information on how to evaluate at the first session of education), respectively. Consistent with this result, in Baraz’s study (2008) clinical evaluation from the nursing students’ perspective was weak (24). One of the objectives of this study was to investigate the relationship between students’ work experience and their perception of monitoring and evaluation in clinical education. So, by increasing of students’ work experience, there was more negative perception to monitoring and evaluation. This could be due to increasing clinical experience, more formal interactions with other nurses, and ongoing assessment by superiors such as nursing supervisors in their work place. The other finding was the difference between male and female nursing students’ perceptions on clinical education. However, the gender differences and its related reasons in how students perceive the clinical education issues should be studied carefully in future research.

One of the limitations of this study is that only students from Tehran university nursing school were studied. So, generalization of the findings should be made with caution. Also, the conservative responses to questions could affect the accuracy of the results. In any case, by creating appropriate conditions for responding to the questionnaire and using non-parametric methods, this limitation was reduced. 

## Conclusion

Clinical education is a very complex process and involves many different dimensions. Given the influence of nursing education on the community health, provision of facilities and revising in evaluation systems of students is recommended. In the present study, there was an attempt to determine the most important barriers and strengths of clinical education from the students’ perspective. In this regard, particular topics in the area of instructor had been confirmed by students. However, the domains of clinical environment, monitoring and evaluation did not show a favorable condition from the students’ perception.

This study describes the issues related to nursing students' perceptions of clinical education. Therefore, determining the students' perspectives can improve the quality of nursing clinical education. It is recommended that studies on nursing students’ perspectives in the years of their academic education (from entering clinical education until graduation) should be conducted using cross-sectional or longitudinal methods and compared with postgraduate period. 
